# Deterministic composite nanophotonic lattices in large area for broadband applications

**DOI:** 10.1038/srep38744

**Published:** 2016-12-12

**Authors:** Jolly Xavier, Jürgen Probst, Christiane Becker

**Affiliations:** 1 Helmholtz-Zentrum Berlin für Materialien und Energie GmbH, Kekuléstr. 5, 12489 Berlin, Germany

## Abstract

Exotic manipulation of the flow of photons in nanoengineered materials with an aperiodic distribution of nanostructures plays a key role in efficiency-enhanced broadband photonic and plasmonic technologies for spectrally tailorable integrated biosensing, nanostructured thin film solarcells, white light emitting diodes, novel plasmonic ensembles etc. Through a generic deterministic nanotechnological route here we show subwavelength-scale silicon (Si) nanostructures on nanoimprinted glass substrate in large area (4 cm^2^) with advanced functional features of aperiodic composite nanophotonic lattices. These nanophotonic aperiodic lattices have easily tailorable supercell tiles with well-defined and discrete lattice basis elements and they show rich Fourier spectra. The presented nanophotonic lattices are designed functionally akin to two-dimensional aperiodic composite lattices with unconventional flexibility- comprising periodic photonic crystals and/or in-plane photonic quasicrystals as pattern design subsystems. The fabricated composite lattice-structured Si nanostructures are comparatively analyzed with a range of nanophotonic structures with conventional lattice geometries of periodic, disordered random as well as in-plane quasicrystalline photonic lattices with comparable lattice parameters. As a proof of concept of compatibility with advanced bottom-up liquid phase crystallized (LPC) Si thin film fabrication, the experimental structural analysis is further extended to double-side-textured deterministic aperiodic lattice-structured 10 μm thick large area LPC Si film on nanoimprinted substrates.

The seemingly counter intuitive impact of *order* as well as *disorder* of aperiodic nanostructures results in achieving tailorable optical properties of nanostructured materials for integrated photonic applications. The dense Fourier spectra of such aperiodic lattice-embedded nanostructured materials could strongly modulate and enable flexible tailoring of the light-matter interaction for broad band nanophotonic applications in comparison to unstructured bulk materials^1^,^2^,^3^,^4^. As the spatial correlations have very important impact on the optical properties of nanophotonic structures^5^, the correlated geometric distribution of nanophotonic lattice points in artificially structured semiconductor thin films could lead to more viable control on light in-coupling as well as light propagation within the semiconductor materials in comparison to its bulk counterpart. In order to ensure technological device practicability with industrial viability, together with the predictive models the specific structural engineering design approaches as well as high throughput large-area fabrication feasibility of high resolution subwavelength nanostructures are unavoidable and highly demanding^2^,^6^,^7^,^8^,^9^. Here we report on easily scalable and tailorable subwavelength scale silicon nanophotonic lattices with effective advanced functional features of ‘*deterministic aperiodic composite photonic lattices*’, from now on called for brevity as ‘*composite lattices*’. They are functionally embedded with designed characteristics of multiple lattice periodicities of conventional photonic crystals and/or in-plane higher order rotational symmetries of common photonic quasicrystals as subsystems in pattern design, hence the denomination ‘*composite lattices*’. By means of advanced nanoengineering, the presented large area composite lattices are deterministically designed and realized functionally similar to conventional two dimensional composite aperiodic lattices, while surpassing the dense distribution of lattice points in real space proportional to the increase in sets of intergrowth elements^10^. Here we report subwavelength-scale silicon nanostructures on nanoimprinted glass substrates in large area up to 4 cm^2^ with advanced functional features of aperiodic composite nanophotonic lattices which are easily tailorable and scalable for the spectral region of interest and application. Though the presented nanostructuring approach is applicable to any semiconductor materials in principle, it is particularly important to silicon, an indirect semiconductor but widely used in integrated broad band applications. As one of the tests of broadband performance of the fabricated Si nanophotonic composite lattices, they are comparatively analyzed with a range of nanophotonic structures with conventional lattice geometries of periodic, disordered random or in-plane quasicrystalline photonic lattices with comparable parameters. The chosen nanoimprinted structures are comparable in terms of fill fraction and spatial reciprocal space relation for an objective reference and scalability. We use a generic Fourier reconstruction method involving selective superposition of components to generate easily tailorable irradiance profiles of composite lattices. By a subsequent thresholding and discretization of lattice points within a chosen mesoscopic supercell with boundary side length typically of 10 to 25 μm, the possible fabrication limitations of the otherwise dense and possibly overlapping spatial distribution of superlattice nanostructures in real space is also surpassed. It is pertinent to be noted that if adopted for substrate structuring using large throughput fabrication processes like nanoimprint lithography, earlier investigated large area deterministic nanophotonic aperiodic lattices with mesoscopic supercell comprising of nanostructures with distributed volumetric basis shapes consisting of very fine and sharp grooves might face a technological challenge. Those structurally fine-featured supercells may get drastically modified during overlaying multi-layered photonic device fabrication processes of standard bottom-up approach^7^,^8^. This eventually may lead to deviation in their expected scattering as well as light trapping performance in addition to undesired increment in dislocation density in the substrate-silicon interfaces in the final device. So in addition to the designed aperiodic geometric nature of the supercell to facilitate rich Fourier spectra, in the presented nanophotonic composite lattices we have also considered the tailorable number of lattice points and discretized motif at individual lattice point which stays intact even during the multilayered deposition. As a proof of concept of this compatibility, in the last section we also present the structural analysis of liquid phase crystallized double-side-textured deterministic aperiodic lattice-structured 10 μm thick large area crystalline silicon film on nanoimprinted substrates. This opens up new vistas in the field of advanced nanoengineering for broadband integrated photonic and plasmonic applications such as colorimetric fingerprints in spectrally tunable biosensors, exotic white LEDs and flat.

panels, slow light integrated chips, structured thin film photovoltaics, novel surface-enhanced Raman scattering nanoplasmonic building blocks, artificially engineered tailorable photonic bandgap materials and efficient nanophotonic test beds for nonlinear light-matter interactions like Anderson localization, and multiple wavelength higher harmonic generation^2^,^3^,^4^,^8^,^11^,^12^,^13^,^14^,^15^,^16^,^17^,^18^,^19^,^20^,^21^.

## Design and computational analysis

**Figure 1:**
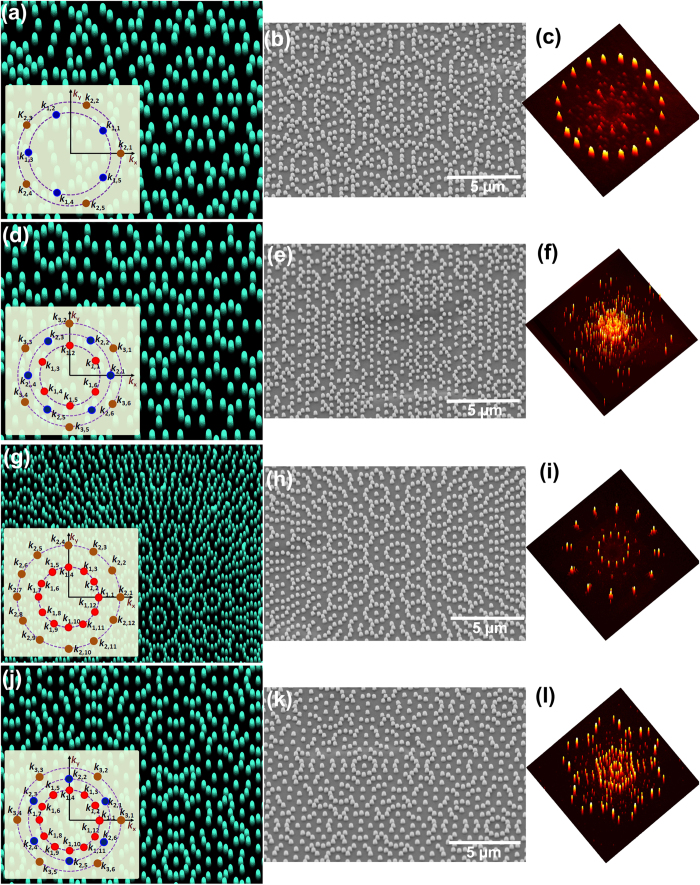
Deterministic aperiodic composite nanophotonic lattices. First column: Part of computed composite lattice structure (Inset: Sets of superposing k-vector component representation in k_x_-k_y_ plane), Second column: Experimental SEM images (40° tilted) of nanoimprinted substrates, Third Column: Experimentally recorded diffraction pattern intensity distribution. (**a**–**c**) PPC_*penta*_ with *s* = 2, *q*_*1*_ = *q*_*2*_ = 5. (**d**–**f**) PPC_*hexa*_ with *s* = 3, *q*_*1*_ = *q*_*2*_ = *q*_*3*_ = 6. (**g**–**i**) PPC_*dodeca*_ with *s* = 2, *q*_*1*_ = *q*_*2*_ = 12. (**j**–**l**) PSC with *s* = 3, *q*_*1*_ = 12, *q*_*2*_ = *q*_*3*_ = 6.

**Figure 2:**
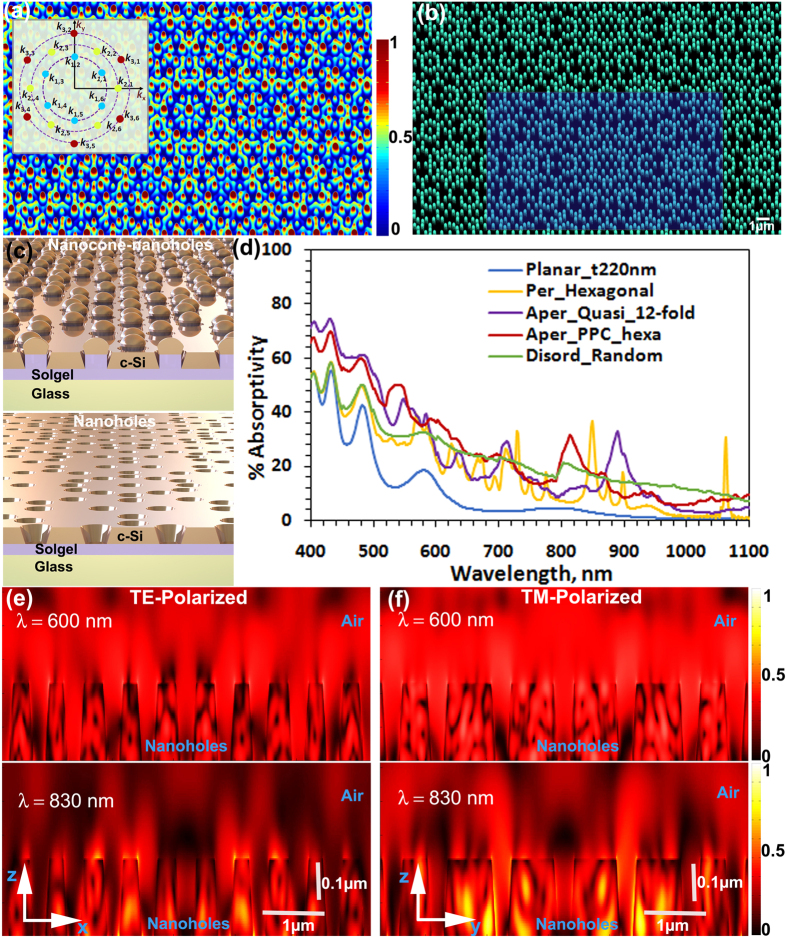
Computational analysis of PPC_*hexa*_ with *s* = 3, *q*_*1*_ = *q*_*2*_ = q_*3*_ = 6. (**a**) Intensity distribution of the basic interference irradiance profile. Inset: Sets of superposing k-vector component representation in k_x_-k_y_ plane. (**b**) Generation of the discretized lattice by tiling the mesoscale supercell shown in blue shadowed region. (**c**) Schematic of respectively nanocone-nanoholes (top) and nanoholes (bottom) (**d**) Computed broadband absorption spectra of nanohole structured c-Si films of thickness = 220 nm. (**e**,**f**) Part of electric field intensity distribution in cross sectional planes along the center of the 10.4 μm × 18.01 μm sized supercell with 741 tapered nanoholes in c-Si thin film.

Here the nanostructured composite lattice is designed by Fourier reconstruction involving the super position of selective plane waves distributed in *s* ≥ 2 sets and subsequent discretization (*see Methods*). Each set has a characteristic length scale and a rotational symmetry *q*_*m*_ (where *m* is from 1 to *s*). In Fig. 1 we show a few examples of composite lattices, classified into two types. The first three rows (Fig. 1a–i) show poly periodic composite (PPC) photonic lattices where the number *q*_*m*_ is same in all sets, starting with a lattice embedded with ten-fold rotational symmetry (*q*_*m*_ = 5), a hexagonal PPC comprised of three sets (*q*_*m*_ = 6) and a PPC with dodecahedral rotational symmetry (*q*_*m*_ = 12). The last row (Fig. 1j–l) shows a poly symmetry composite (PSC) photonic lattice where the number *q*_*m*_ is not same in all sets, combining both in-plane quasicrystalline 12-fold rotational symmetry structures as well as a mesoscopic hexagonal order. The insets in the first column of Fig. 1 show the sets of *k*-vector components giving the characteristic representation of each design, scanning electron microscopic (SEM) images of experimentally realized nanoimprinted glass substrates and their respective far field diffraction pattern intensity distributions are shown in the second and third columns. In principle any practically viable arbitrary number of rotational symmetries can be embedded given the complexity involved in the generated aperiodic pattern. For brevity, conventional periodic and in-plane qauasicrystalline photonic lattices can be considered here as subgroups within this broad class of photonic lattices when *s* = 1 and the respective number *q* point to their fundamental rotational symmetry. To picturize the details of our approach we closely consider one of the above cases, PPC_*hexa*_ (*see also Methods*), where *s* = 3 and *q*_*1*_ = *q*_*2*_ = *q*_*3*_ = 6 (inset of Fig. 2a) with absolute amplitude strengths of the components in respective set as 0.5:0.25:1.

In the subsequent sections we make computational as well as experimental comparative analysis of this composite photonic lattice with periodic, in-plane quasicrystalline as well as disordered photonic lattices with comparable fill fraction. The considered radial distance from the origin of the components in set 1 corresponds to that of the first order Fourier components of a hexagonal lattice with real space lattice constant *a*_*1*_ = 1000 nm whereas for the components in set 2 and 3, it is respectively *a*_*2*_ = 800 nm and *a*_*3*_ = 600 nm. The mesoscale supercell (shaded region in Fig. 2b) lateral dimensions are respectively *a*_*PPC*_ = 10.4 μm and *b*_*PPC*_ = 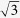
*a*_*PPC*_ = 18.01 μm, such that the complementary edges keep the rotational symmetry of the basic supercell intact even across neighboring tile boundaries. This supercell with 741 lattice points is tiled to get the large area composite lattice. The individual rod diameter of 236 nm is calculated such that same fill fraction is obtained comparable to periodic, quasicrystalline and random disordered lattices used in the present study. Given the geometrical and basis structural advantages of the tailorable composite lattices, they could very well be designed to simultaneously couple the photon flux in the UV, and trap the visible as well as the infrared region either for broad band applications or for a specific spectral range. In par with a standard silicon on insulator film, we chose one of the samples under consideration here a 200 nm thick Si thin film structured with tapered nanoholes on a glass substrate with a slanting angle of 17° as observed in the experimental scanning images. The tapered nanohole bottom diameter was chosen to be 196 nm where a 15% shrinkage in the solgel nanoimprinted lattice rod diameter is considered as indicated in the experimental analysis. Apart from a planar reference c-Si thin film, for the computational as well as the subsequent experimental comparative analysis in the present study we included c-Si thin films structured with periodic hexagonal lattice, in-plane 12-fold symmetry quasicrystal, and disorder-induced random lattice (Fig. S2 Supplementary material). Using a 3D Finite Difference Time Domain (FDTD) Maxwell’s equation solver (Lumerical) we computed the broadband absorption spectra as given in Fig. 2d. As it is seen, the aperiodic lattices show an enhanced integrated absorption spectrum in the broad band range. The broad band light coupling as well as efficient light trapping of the designed PPC_*hexa*_ structure is further visualized by computing the cross sectional field intensity distribution of the aperiodic lattice while a plane wave is incident from above. The 3D FDTD computer simulation of the electric field intensity distribution for two wavelength regions at λ = 600 nm as well as at λ = 830 nm for TM and TE incident polarizations is given in Fig. 2e and f showing the high field in-coupling and confinement which in turn leads to overall enhanced absorption (Fig. 2d), which will be further proved in the experimental analysis later in this study.

**Figure 3:**
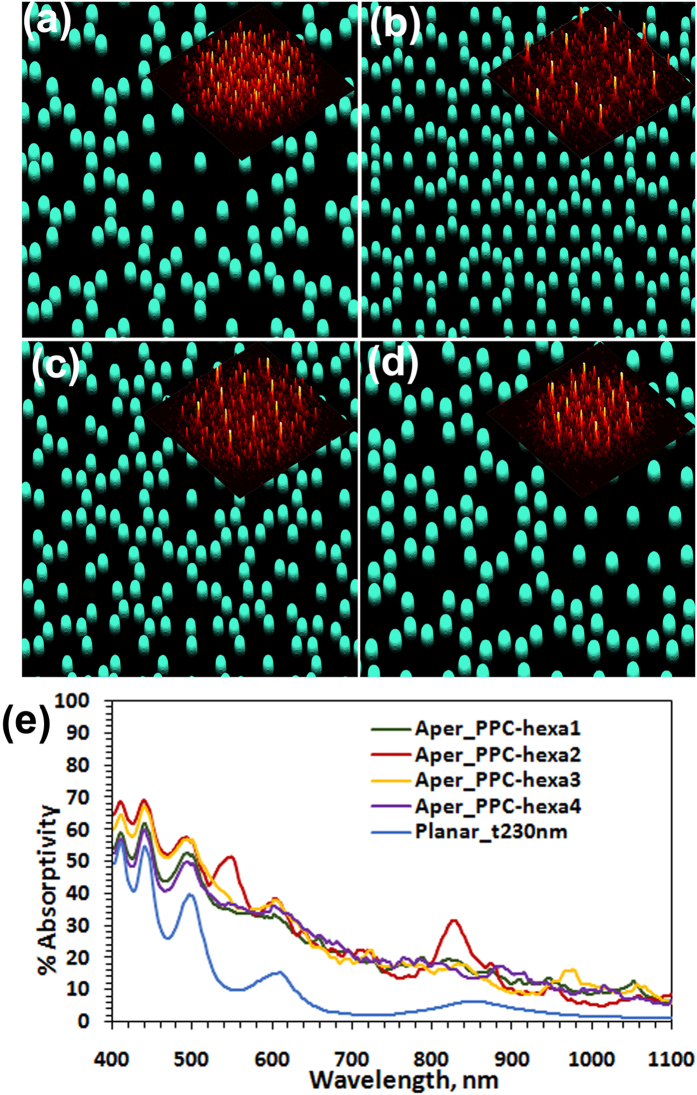
Nanoengineering the lattice point distribution. Tailoring the lattice point distribution of PPC_*hexa*_ composite lattice with *s* = 3, *q*_*1*_ = *q*_*2*_ = *q*_*3*_ = 6 by tuning the ratio of the absolute amplitude strengths of the components in each set. Inset: Resultant Fourier spectrum. (**a**) PPC_*hexa-1*_ with ratio 1:1:1. (**b**) PPC_*hexa-2*_ with ratio 0.5:0.25:1. (**c**) PPC_*hexa-3*_ with ratio 0.25:1:0.5. (**d**) PPC_*hexa-4*_ with ratio 1:0.5:0.25. (**e**) Computed broadband absorption spectra of nanoholes-structured Si thin films (Si thickness = 230 nm).

In Figs 3 and S3 (see Supplementary material) we give our computational results for the present aperiodic PPC_*hexa*_ by tuning the amplitude strength of the three sets of k-vector components whereby the strength of a particular intergrowth pitch could be tailored individually for varying spectral applications without affecting the inherent lattice rotational symmetry or the basis pattern at the lattice points. Given the thickness of the Si film, as seen in Fig. 3e the absorption spectral response to a range can be tailored without changing the embedded rotational symmetry of the aperiodic lattice. While PPC_*hexa-2*_ and PPC_*hexa-3*_ respond well to the shorter wavelength range, PPC_*hexa-4*_ absorbs comparatively well the longer wavelengths. For the present case, PPC_*hexa-2*_ has an efficient broadband absorption spectra among all.

## Experimental Results and Discussion

**Figure 4:**
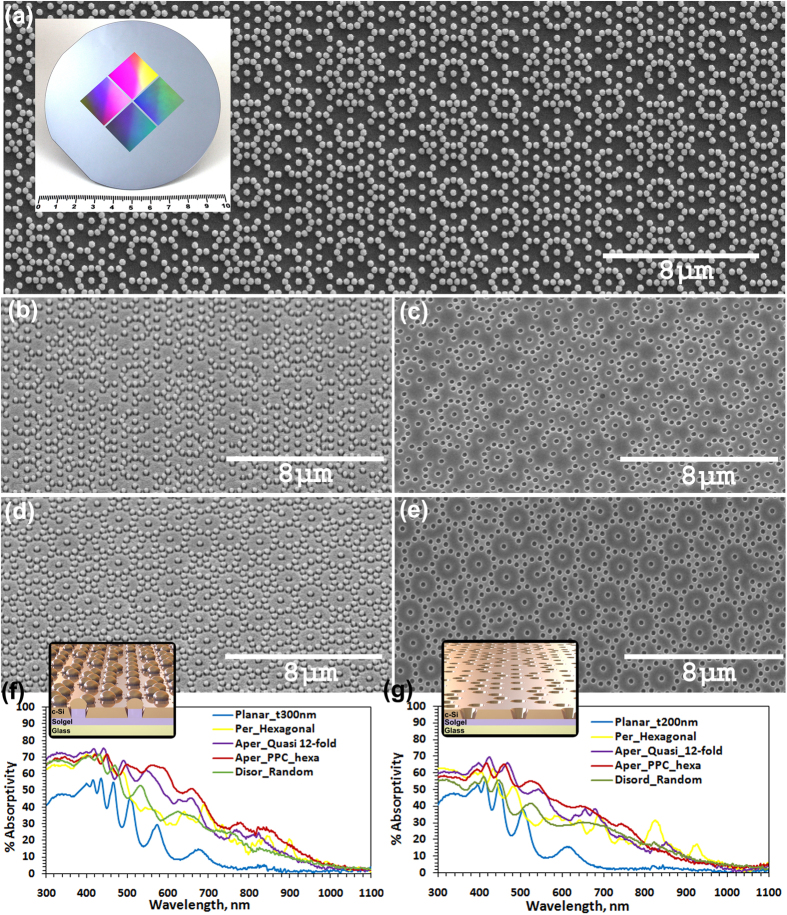
Experimental analysis of deterministic aperiodic nanostructured c-Si thin films. (**a**) SEM image of the fabricated large area PPC_*hexa*_ composite lattice structured thin film with Si nanodomes (prior to crystallization) on nanoimprinted glass substrate (Scale bar: 8 μm). [Inset: Photograph of one of the Si wafers patterned via e-beam lithography, which is designed and fabricated as master wafer for nanoimprinting (Scale bar: 10 cm).] (**b**,**c**) SEM images of the PPC_*hexa*_ composite lattice structured c-Si thin film respectively textured with nanocone-nanoholes (Images are tilted by 40°) (**b**) and nanoholes (**c**). (**d**,**e**) SEM images of the in-plane 12-fold symmetry quasicrystal-structured c-Si thin film respectively textured with nanocone-nanoholes (**d**) and nanoholes (**e**). (**f**,**g**) Experimental broadband absorption spectra respectively for the c-Si thin films textured with nanocone-nanoholes (**f**) and nanoholes (**g**). Inset: Schematic representation of the respective layered structure.

Our approach for large area deterministic fabrication (*see Methods*) of diverse composite photonic lattices, in-plane quasicrystals, periodic lattices and the disordered random lattices involves state of art high resolution e-beam lithography for the one-time master wafer fabrication and subsequent large throughput as well as cost effective nanoimprint lithography for highly resolved large area nanostructures (Fig. 1b) tailorable for diverse applications^22^,^23^. A photographic image of one of the designed and fabricated master wafers is given in inset of Fig. 4a. Subsequently nanoimprinted glasses featuring sol-gel rods at each discrete lattice point were used as substrate for the fabrication of silicon nanocone-nanoholes (NCNH) as well as tapered nanohole arrays (NH) (*see Methods*). In Fig. 4a, a SEM image of Si nanodomes (prior to crystallization) with PPC_*hexa*_ geometry is given. Further we show, after a polysilicon wet etch process (*see Methods*), the subsequently fabricated PPC_*hexa*_ and 12-fold rotational symmetry NCNH-embedded Si thin films with 300 nm Si deposition thickness (Fig. 4b and d respectively) and Si film of 200 nm thickness structured with tapered NH (Fig. 4c and e respectively) on nanoimprinted glass substrates. In Fig. S4 (see Supplementary material) we give the SEM images of a few fabricated comparable Si thin films structured with NCNH and tapered NH with periodic, in-plane quasicrystalline as well as disordered random lattice geometry realized through the same approach.

In view of varied applications in biosensing^11^ and light harvesting^4^ etc., an experimental quantitative transmission analysis of the studied nanostructured Si thin films on nanoimprinted substrates is carried out. The observed absorption spectral analysis is given in Fig. 4f and g. The 3D visualization schematics of the material layers and the lattice point motifs of NCNH and tapered NH are respectively given in the inset of Fig. 4f and g. The experimental absorption analysis of the studied samples show the enhanced broadband performance of aperiodic nanostructures with tailored rich Fourier spectra of PPC lattices and in-plane quasicrystalline lattices for improved light channeling into the absorbing medium which also enables enhanced quasi guided light propagation within the material leading to better light trapping within the material. In order to analyze the geometrical features of the studied nanostructured thin film and its impact on the optical performance of the structured thin films, we neither used any antireflection coating nor any back reflectors in the present study. The use of such material layers for a given application naturally improves further the absorption in the Si thin films^17^,^23^. For a quantitative analysis, we also estimated the maximum achievable short circuit current density *j*_sc,max_ (*see Methods*) as summarized in Table 1. From Fig. 4f - g and Table 1, for the studied two different cases (NHs in 200 nm thin film and NCNHs in 300 nm thin film) it is observed that a +18.36% and +30% relative higher *j*_*sc,max*_ is estimated for artificially engineered Si nanostructures with the lattice geometry of PPC_*hexa*_ compared to the corresponding value of the periodic hexagonal lattice under investigation and the same trend of enhancement is observed in comparison to the investigated disordered random lattice too. Whereas these enhancements in comparison to a planar reference were respectively +175.3% and +189.8%. For the aperiodic in-plane 12-fold symmetry quasicrystal structured samples with NH and NCNH respectively these enhancements in *j*_*sc,max*_ were +10.5% and +14.28% in comparison to hexagonal lattice. It can be observed from the optical absorption spectra (Fig. 4f and g) that the increased broadband absorption in the studied c-Si thin films is due to strong suppression of reflection losses in the lower wavelength range (λ < 500 nm) and enhanced absorption in longer wavelength range (650 nm < λ < 900 nm). The engineered composite aperiodic nanostructures with combined functional geometric features belonging to multiple lattice periodicities as in PPC_*hexa*_ of the present study could effectively couple the incident light into quasi-guided modes of the silicon film of studied thickness. Moreover it has also been observed that the aperiodic structures with higher rotational symmetry lattice geometries also show angle-independent broad band absorption of light^15^,^24^. A non-zero absorption around 1100 nm by all the studied samples could be attributed to the residual absorption by the underlying solgel layer as have been observed earlier also in Si thin films in nanoimprinted substrates^25^. In a nutshell, our experimental results strongly indicate that the presented tailored composite and quasicrystalline aperiodic lattice-embedded artificially nanoengineered materials are excellently suited for broadband light coupling and absorption enhancement in ultrathin nanophotonic integrated devices which can be easily optimized suiting to the application requirement. They exploit in a unified manner the resonant optical properties of periodic lattices in multi-spectral regions enabling the broadband features of the efficiently tailorable disordered nanophotonic structures in a versatile manner. Given the demonstrated design, the fabrication and analysis of composite lattices which could also be further optimized and the presented approach gives the generic freedom to design and fabricate diverse deterministic aperiodic lattices. The designed aperiodic lattices can be modified easily with tailorable set of design parameters suiting to the desired different thin film thicknesses and materials befitting to various photonic integrated applications and spectral regions of interest. While the abstract design of complex nanostructures is possible to any extent, it is also pertinent to taken care that the designed structures are also compatible with large area high resolution fabrication processes with the realistic industrial viability and mass production retaining the designed desired geometrical and basis structural features. Considering the presence of structured multiple layers and the interfaces in the actual integrated device fabrication with multiple thin film layer interfaces, rigorous light-matter interaction analyses and optimizations on light propagation and light trapping are also to be taken into consideration in addition to the Fourier reconstruction-enabled substrate structuring analysis which we have primarily considered here to show the feasibility and advantage of the presented approach.

**Figure 5:**
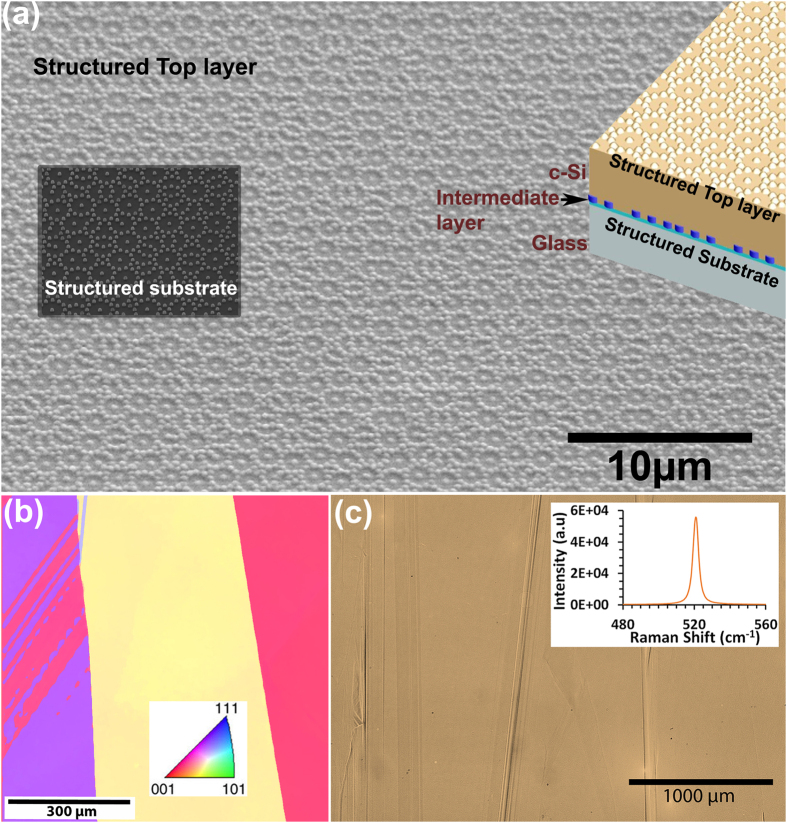
Deterministic double side aperiodic lattice-textured c-Si thin films. (**a**) SEM image of the top surface of the layered sample with laser beam-assisted liquid phase crystalized Si film of 10 μm thickness. Left inset: Structured substrate at the bottom. Right inset: Schematic of Glass (bottom most)-Solgel-SiO_x_-Si-SiN_x_ layered sample. (**b**) One of the EBSD surface orientation maps of double side aperiodic lattice-structured LPC Si thin film prior to SiN_x_ deposition. The color coding of the different crystallographic orientations is depicted in the inset. (**c**) Optical microscope large area image of the top surface. Inset: First-order Raman scattering at room temperature from double side aperiodic lattice-structured LPC Si thin film.

Extending further, as a proof of concept of compatibility with advanced bottom up liquid phase crystallized (LPC) Si thin film fabrication, we fabricate and do the detailed structural analysis of deterministic aperiodic double-side textured 10 μm thick Si films with enhanced material quality via laser beam crystallization. The geometrical as well as discrete motifs of the deterministic aperiodic lattice with subwavelength features is well maintained even after multiple layer depositions (*see Methods*). In Fig. 5 we show the double side aperiodic lattice textured LPC Si thin film realized through the present approach. The left inset of Fig. 5a gives the SEM image of the bottom structured substrate on which the subsequent multi layers are deposited, while a schematic of the layered structure is given in the right inset. Further material structural quality analysis are done using electron backscatter diffraction (EBSD) microstructural-crystallographic orientation mapping (Fig. 5b), optical microscope large area imaging (Fig. 5c) and Raman scattering spectral imaging (Fig. 5c inset). Figure 5b gives one of the EBSD surface orientation mappings, exhibiting elongated large grains of millimeters range, of a LPC c-Si thin film sample with a 100 nm SiO_x_ intermediate layer on nanoimprinted glass substrate. The structural characterizations verify the material structural quality of the deterministic aperiodic structured 10 μm thick LPC Si films with well-maintained basis structural features. If needed the lattice basis structuring can be even further tailored with ease from columnar to smoothed hills in order to tune the electrical material quality of c-Si thin film for varied applications without compromising the designed aperiodic geometrical features. Moreover, our generic approach for nanoengineered deterministic aperiodic lattices with rich Fourier spectra offers a great flexibility of optimization through tailoring their lattice geometry, basis shape, and spectral response range to match to the desired different thin film thicknesses, material of interest and for intended nanophotonic applications. The industrial application-viable and standard bottom-up fabrication technologies-compatible approach which we have demonstrated is envisaged to suit well for broadband integrated nanophotonic devices which demands large throughput and high resolution structured semiconductor materials with advanced tailorable features in nanoscale^1^,^2^,^3^,^4^,^5^. Due to the inherent ability to boost broad band light matter interaction, the presented subwavelength scale deterministic nanophotonic lattices with precise tailorable structural features are envisaged to be appealing option for novel integrated nanophotonic applications^2^.

## Methods

### Design approach

Irradiance profile for various in-plane quasicrystalline photonic lattice structures could be computed by Fourier reconstruction resulting from the designed superposition of plane waves^26^. For the subwavelength nanophotonic structures, the discreteness of the neighboring lattice points are strongly affected as the number of *k*-vector components are increased^7^,^26^. It will be very critical when the components are distributed in multiple sets with varying radial distance from the origin in the *k*_*x*_-*k*_*y*_ Fourier plane. A generalized expression used here to compute the irradiance profile for a desired subwavelength composite photonic lattice structure by Fourier reconstruction resulting from the superposition of 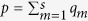 plane waves linearly polarized in same direction and distributed in *s* sets is given by,


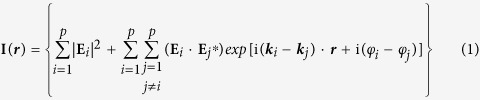


where, ***E***_*i*_, ***k***_*i*_, ***r*** and *φ*_*i*_ are respectively the complex amplitudes, the wave vectors, the position vector and the initial phase. Each set is comprising of *q*_*m*_ (where *m* is from 1 to *s*) azimuthally equidistant components in the *k*_*x*_-*k*_*y*_ Fourier plane. The components in a given set are radially equidistant from the origin designed as per the desired neighbor distance of a constituent lattice structure to be embedded within the composite lattice. The *k*_*m,n*_ component in k-vector diagrams (inset of Fig. 1) represents the *n*^th^ component in *m*^th^ set. The value of *q*_*m*_ in each set basically defines its contributing fundamental rotational symmetry within the composite lattice. With reference to the k-vector representation shown in the inset of Fig. 2a, for the case of PPC_*hexa*_ we used *p* = 18 where *s* = 3 and *q*_*1*_ = *q*_*2*_ = *q*_*3*_ = 6. The radial distance from the origin of the components in the first set corresponds to that of the first order Fourier components of hexagonal lattice with real space lattice constant *a*_*1*_ = 1000 nm whereas the corresponding values for components in second and third sets respectively are *a*_*2*_ = 800 nm and *a*_*3*_ = 600 nm. This gives a means of combining the functional features of three effective pitches within a composite photonic lattice, but at the mean time the real space spatial constraints of accommodating multiple periodicities is alleviated. Further, the strength of amplitude of waves in each of these three sets can be tailored independently and in the present case we have chosen a ratio 0.5:0.25:1. Tuning this ratio is an additional degree of freedom to tailor the composite PPC (Fig. 3). Here the initial phase of all of them are considered to be zero where tuning the same obviously gives yet another possibility to tailor the photonic lattice structures^26^. Moreover, unlike the interference experimental approaches, here the easily viable amplitude modulation doesn’t lead to any intensity loss as the interference patterns are only virtually involved in the design so the approach doesn’t necessitates the phase modulation. The amplitude modulation is easier and versatile to realize diverse intensity patterns, so we have implemented the same in the present case. Moreover there are no concrete optical elements being involved in the nanostructure pattern formation in subwavelength scale where the beam intensity loss is to be considered unlike the case of interference lithography. The computed aperiodic lattice pattern is quite large in spatial extension which makes it inappropriate to realize such complex lattices by standard high resolution nanofabrication methods. So in a subsequent step, a large rectangular supercell is chosen from the computed pattern. Next, the pattern within the supercell is discretized by applying a certain threshold to the continuous function, determining the geometric centroids of the area above the set threshold and subsequently tiling the supercell to generate a large area composite lattice (Fig. 2b). Here, we chose a threshold intensity as well as bright spot perimeter equal to 0.3 times of peak values within the supercell. By tuning these threshold values, the number of discrete lattice points within the supercell can be easily tuned without affecting the rotational symmetry properties of the generated lattice to a great extent. The discretized supercell should contain sufficient number of lattice points permitting a discrete Fourier pattern with high finesse (Fig. S1). At the mean time the dimensions of the supercell are chosen to be compatible enough both for 3D computational analysis as well as standard high resolution fabrication approaches such as electron-beam lithography. The lateral dimensions of the supercell (blue shaded region in Fig. 2b) of this designed PPC_*hexa*_ are respectively *a*_*PPC*_ = 10.4 μm and *b*_*PPC*_ = 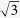
*a*_*PPC*_ = 18.01 μm and have complementary edges keeping the rotational symmetry of the basic supercell intact over the whole tiled region. This supercell has 741 lattice points and is subsequently tiled to realize the large area composite photonic lattice. Fig. S1 gives an example on choice of supercell size and its effect on the resultant Fourier pattern, where a 10-fold in-plane quasicrystal lattice supercell is optimized with lateral dimensions of 9.4 × 11.05 μm^2^ (see Supplementary material). In Fig. S2 (see Supplementary material) we outline the wave design k-vector diagrams, the nanoimprinted substrates and the respective far-field diffraction patterns of large area periodic square and hexagonal lattices as well as in-plane quasicrystals with 10-fold and 12-fold rotational symmetries. For a fair comparison of the impact of different lattice geometries it is needed to realize nanoimprinted lattice structures for all the considered photonic lattices with equal fill fractions but at the same time discrete and non-overlapping basis structure at each lattice point within the supercell. So we have chosen a corresponding lattice constant of 700 nm of the simplest 2D periodic structure with square lattice as the reference lattice constant considering the fact that comparable aperiodic lattices would result in an even smaller nearest neighbor distance approaching 400 nm. All the lattices given in Fig. S2 are designed such that they all have equal fill fraction by taking care of their rod diameters with respect to the number of rods in a given supercell. Moreover they all are designed with comparable lattice constant or nearest neighbor distance indicated by their equal radial distance of the prominent diffraction order from the origin giving an equivalent reciprocal space relation between one another. We also designed a supercell (~14.5 μm × 14.0 μm) of a randomly disordered lattice which was fundamentally generated by in-plane perturbing the in-plane Cartesian coordinates of the lattice points of the corresponding periodic hexagonal lattice with *a*_*hex*_ = 808 nm used in our study by an amount chosen randomly from a uniform distribution of values between 0 and 0.3 *a*_*hex*_.

### Nanostructured master wafer by electron-beam lithography

Structured silicon master wafers were prepared by electron beam lithography, mask inversion by a lift-off process and final reactive ion etching. Each of the presented photonic lattice pattern was exposed by a 100 kV Vistec EBPG5000+ES onto an area of 2.0 cm × 2.1 cm on a silicon substrate coated with a 160 nm thick layer of positive electron beam resist ZEP520A (ZEON Corp.). After development in hexyl acetate and rinsing in isopropyl alcohol, the structured resist was coated with a 30 nm thick nickel layer, which served as etch mask after resist removal in a lift-off procedure in dimethyl formamide. Finally, the silicon was etched 370 nm deep in a highly anisotropic reactive ion etching process (etching gases SF_6_, C_4_F_8_ and O_2_) and the nickel mask was subsequently removed in hydrochloric acid.

### Nanoimprinted Glass substrates

After a short oxygen plasma pre-treatment, the master wafer with nanostructures is coated with a thin anti-sticking layer via vapor deposition of (tridecafluoro-1, 1, 2, 2-tetrahydrooctyl) trichlorosilane short F13-TCS (CAS: 78560-45-9, ABCR GmbH) in a vacuum oven. As per the requirement, for high resolution nanoimprint process we prepared composite poly-(dimethyl) siloxane (PDMS) stamps or else conventional soft PDMS stamps were used. Composite PDMS (c-PDMS) stamps are composed of two layers: a thin hard PDMS (h-PDMS) layer supported by a thick soft PDMS (s-PDMS) layer. For the c-PDMS stamp preparation^27^, first the h-PDMS is made by mixing step by step 3.4 g of a vinylsiloxane prepolymer (CAS 67762-94-1, ABCR GmbH) with 0.016 g of platinum-divinyltetramethyldisiloxane as catalyst (CAS 68478-92-2, ABCR GmbH), 0.1 g of 2,4,6,8-tetramethyl-2,4,6,8-tetravinylcyclotetrasiloxane (CAS 68478-92-2, Sigma-Aldrich Corp.) as modulator and 1 g of a hydrosiloxane prepolymer (CAS 68037-59-2, ABCR GmbH). To this mixture 2 g of Toluene was blended as solvent. This final h-PDMS mixture was spin coated directly on the master wafer and left for 1 hour and subsequently cured for 1 hour at 60 °C. Now, the s-PDMS was prepared by mixing the poly-(dimethyl) siloxane and its catalyst (PDMS, Elastosil RT A/B 601 from Wacker) with a ratio of 9:1. On the Master wafer with the thin of h-PDMS layer on top, the degassed s-PDMS is poured and subsequently slowly cured in an oven at 35 °C for overnight. The final c-PDMS stamp can then be gently peeled off from the master and used as a mold for the replication process. The sol-gel (Philips) is now spin coated on cleaned glass substrates (Corning Eagle). The PDMS stamp is placed on the sol-gel coated glass substrate and cured with a UV (λ = 400 nm) lamp for about 6 minutes. After UV-curing, the PDMS stamp is carefully peeled off the substrate leaving the imprinted nanostructures corresponding to the master wafer. After a quick post-bake for 8 minutes at 100 °C, the nanoimprinted glass substrates are further thermally annealed at 600 °C for an hour in order to ensure the thermal stability of the sol-gel textured glass substrate by effusing out the remaining organic residues.

### Fabrication of structured silicon thin films

For the fabrication of nanocone-nanoholes (NCNH) and nanoholes (NH) structured Si thin films, a Si layer of the desired thickness is deposited by electron-beam evaporation in amorphous phase on the cleaned nanoimprinted glass substrates at a substrate-temperature of 300 °C. Subsequent thermal annealing at 600 °C for several hours in a tube furnace within an N_2_ atmosphere results in solid phase crystallization of the silicon film aside from the columnar substrate features. The still amorphous Si parts around the columns are removed by wet-chemical etching in an etch solution consisting of concentrated HNO_3_ (65%, 30 parts), concentrated H_3_PO_4_ (85%, 10 parts), HF (50%, 1 part) and H_2_O (43 parts) resulting in nanocone-nanoholes^28^. The nanoholes are realized by mechanically detaching the sol-gel columns by abrasion.

For the fabrication of the presented double-side textured liquid phase crystallized Si thin films, first a SiO_x_ diffusion barrier layer of around 100 nm thickness is deposited on the cleaned nanoimprinted glass substrates by physical vapour deposition using a reactive magnetron sputtering system. Subsequently the desired thickness of nominally intrinsic nanocrystalline Si was deposited by e-beam evaporation at a substrate-temperature of 600 °C. Subsequently, a 250 nm thick SiO_x_ capping layer was deposited on top of the layer stack by reactive magnetron sputtering. This capping layer prevents the surface of the Si melt from levelling out during crystallization and thus allows maintaining the initial topography of the deposited layer akin to that of the nanoimprinted substrate texturing^29^. Moreover, this cap layer prevents from the dewetting of the liquid Si thin film as well. Then the samples were crystallized using a line shaped cw laser (λ = 808 nm ± 10 nm) from LIMO GmbH^30^. The used optical intensity for the crystallization process was around 3.58 kW/cm^2^. Crystallization was performed at a substrate temperature of 600 °C and by moving the samples under the laser beam with a velocity of 11 mm/s. Further, the SiO_x_ capping layer at the surface was removed using a 5% HF solution as etchant in a wet etching process resulting in a double side textured Si thin film with well-maintained structural features of the nanoimprinted substrate.

### Absorption measurement and short circuit current density calculation

The absorption spectra of the nano-patterned crystalline silicon thin films were measured inside an integrating sphere of a PerkinElmer1050 optical spectrometer with wavelength resolution of 2 nm. Samples were tilted by 8 degrees to ensure that the specularly reflected light does not escape through the entrance port. The maximum achievable short circuit current density *j*_sc,max_ is calculated by,


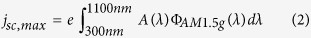


with elementary charge *e*, absorption *A*(λ) and Φ_AM1.5g_ (λ) the photon flux of the AM1.5 g solar irradiance spectrum. It represents the upper limit of the current density of photovoltaic devices at zero voltage at illumination with the global solar irradiance spectrum AM1.5 g and serves as figure of merit for integrated absorption. Here, it is assumed that each photon absorbed in the spectral range from 300 to 1100 nm in the nanostructured silicon film contributes to the solar cell current and no electrical losses occur, e.g. by recombination.

## Additional Information

**How to cite this article**: Xavier, J. *et al*. Deterministic composite nanophotonic lattices in large area for broadband applications. *Sci. Rep.* **6**, 38744; doi: 10.1038/srep38744 (2016).

**Publisher's note:** Springer Nature remains neutral with regard to jurisdictional claims in published maps and institutional affiliations.

## Supplementary Material

Supplementary Information

## Figures and Tables

**Table 1:** Summary of broadband absorption characteristics of investigated structured c-Si thin films.

Lattice structure	Planar	Hexagonal	12-fold quasi	PPC_*hexa*_	Disordered random
Nanoholes (Silicon reference thickness = 200 nm)					
J_sc max_ (mA/cm^2^)	4.7	10.9	12.1	12.9	10.4
Enhancement (versus hexagonal)	−56.9%	—	**+11%**	**+18.3%**	−4.6%
Enhancement (versus planar)	—	+131.9%	**+157.4%**	**+174.5%**	+121.3%
Nanocone-nanoholes (Silicon reference thickness = 300 nm)					
J_sc max_ (mA/cm^2^)	5.6	12.5	14.4	16.3	12.3
Enhancement (versus hexagonal)	−55.2%	—	**+14.3%**	**+30.4%**	−1.6%
Enhancement (versus planar)	—	+123.2%	**+157.1%**	**+191.1%**	+119.6%
