# Supplementary material for: Deterministic composite nanophotonic lattices in large area for broadband applications
Source: Sci Rep. 2016 Dec 12;6:38744. doi: 10.1038/srep38744 (PMC5150530; doi:10.1038/srep38744)

Deterministic composite nanophotonic lattices in large area for broadband applications

Jolly Xavier*†, Jürgen Probst*, and Christiane Becker

Helmholtz-Zentrum Berlin für Materialien und Energie GmbH, Kekuléstr. 5, 12489 Berlin, Germany

†Present Address: Max Planck Institute for the Science of Light, Guenther-Scharowsky-Str.1, 91058 Erlangen, Germany

Supplementary Material

Figure S1 | Lattice point distribution within the chosen supercell of studied 10-fold symmetry transversely quasicrystalline lattice and its effect on the finesse of the resultant Fourier spectral peaks. Inset on top left: ***k***-vector component representation in k_x-k_y plane. (a)-(f) Discretized super cell (top) and its respective Fourier spectra (bottom). Inset: Original irradiance profile. (a) Supercell with 30 lattice points. (b) Super cell with 40 lattice points. (c) Super cell with 62 lattice points. (d) Super cell with 101 lattice points. (e) Super cell with 107 lattice points. (f) Super cell with 271 lattice points (used for subsequent fabrication).

Figure S2 | Fabricated large-area periodic, and transversely quasicrystallographic photonic lattices. The photonic lattices have been designed such that first prominent Fourier order is diffracted at the same angle from the origin (blue scale bar). The diameters of the nanopillars have been calculated for a resultant equal fill fractions for all the studied lattice structures. First column: ***k***-vector component representation in k_x-k_y plane. Second column: SEM images of photonic lattice nanoimprinted substrates with equal fill fraction Third Column: Experimentally recorded diffraction pattern while a 532 nm laser beam is incident on the nanoimprinted sample. (a)-(c) Periodic square photonic lattice. (d)-(f) Periodic hexagonal photonic lattice. (g)-(i) Transversely 10-fold symmetry quasicrystalline photonic lattice. (j)-(l) Transversely 12-fold symmetry quasicrystalline photonic lattice.

Figure S3 | Computed lattice point distribution of PPC_{hexa} composite lattice with s = 3, $q_1 = q_2 = q_3 = 6$ while tuning the ratio of the absolute amplitude strengths of the components in each set. Inset: Resultant Fourier spectrum (a) PPC_{hexa-2} with ratio 0.5:0.25:1. (b) PPC_{hexa-3} with ratio 0.25:1:0.5. (c) PPC_{hexa-4} with ratio 1:0.5:0.25. (d) PPC_{hexa-5} with ratio 0.25:0.5:1. (e) PPC_{hexa-6} with ratio 1:0.25:0.5. (f) PPC_{hexa-7} with ratio 0.5:1:0.25.

Figure S4 | SEM images of the fabricated large area c-Si thin film structured respectively with nanocone-nanoholes (Left column) (40° tilted images) and nanoholes (Right column), with lattice symmetry of (a)-(b) Periodic hexagonal lattice, (c)-(d) Transversely 10-fold symmetry quasicrystalline lattice, and (e)-(f) Disordered random lattice.

Figure S1

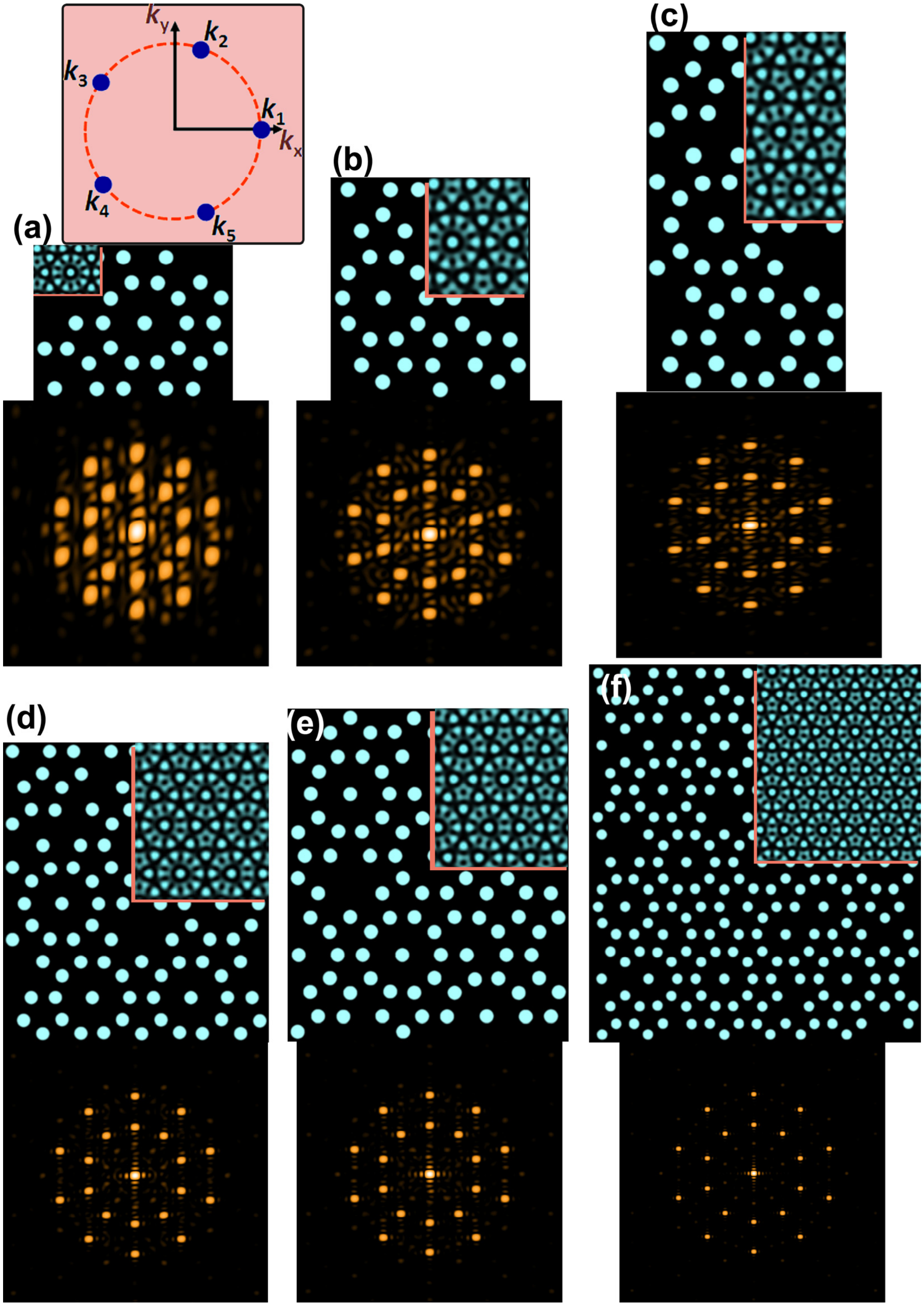

Figure S2

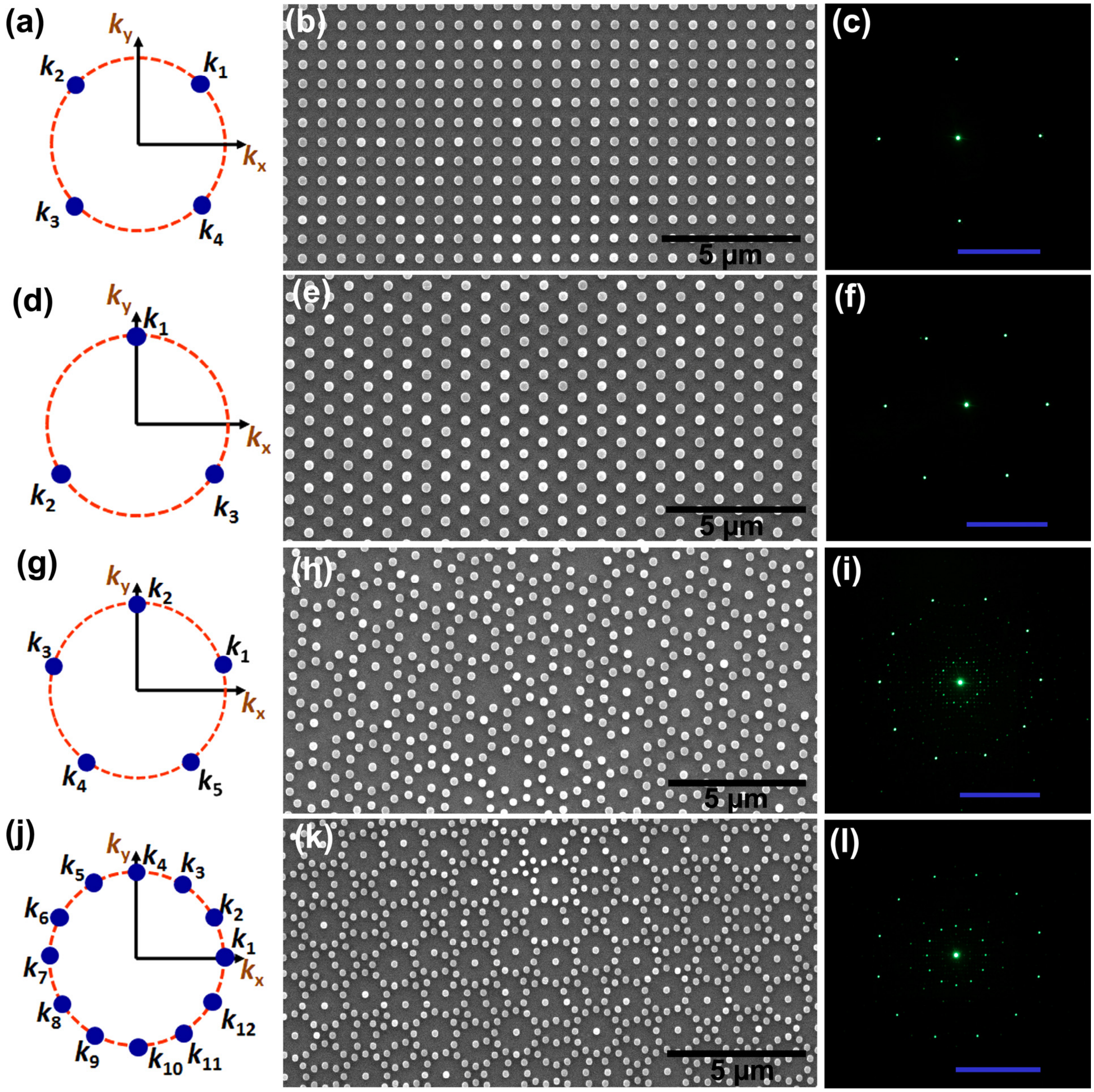

(a)
k_y
k_x
k_1
k_2
k_3
k_4
(b)
5 μm
(c)
(d)
k_y
k_x
k_1
k_2
k_3
(e)
5 μm
(f)
(g)
k_y
k_x
k_1
k_2
k_3
k_4
k_5
(h)
5 μm
(i)
(j)
k_y
k_x
k_1
k_2
k_3
k_4
k_5
k_6
k_7
k_8
k_9
k_{10}
k_{11}
k_{12}
(k)
5 μm
(l)

Figure S3

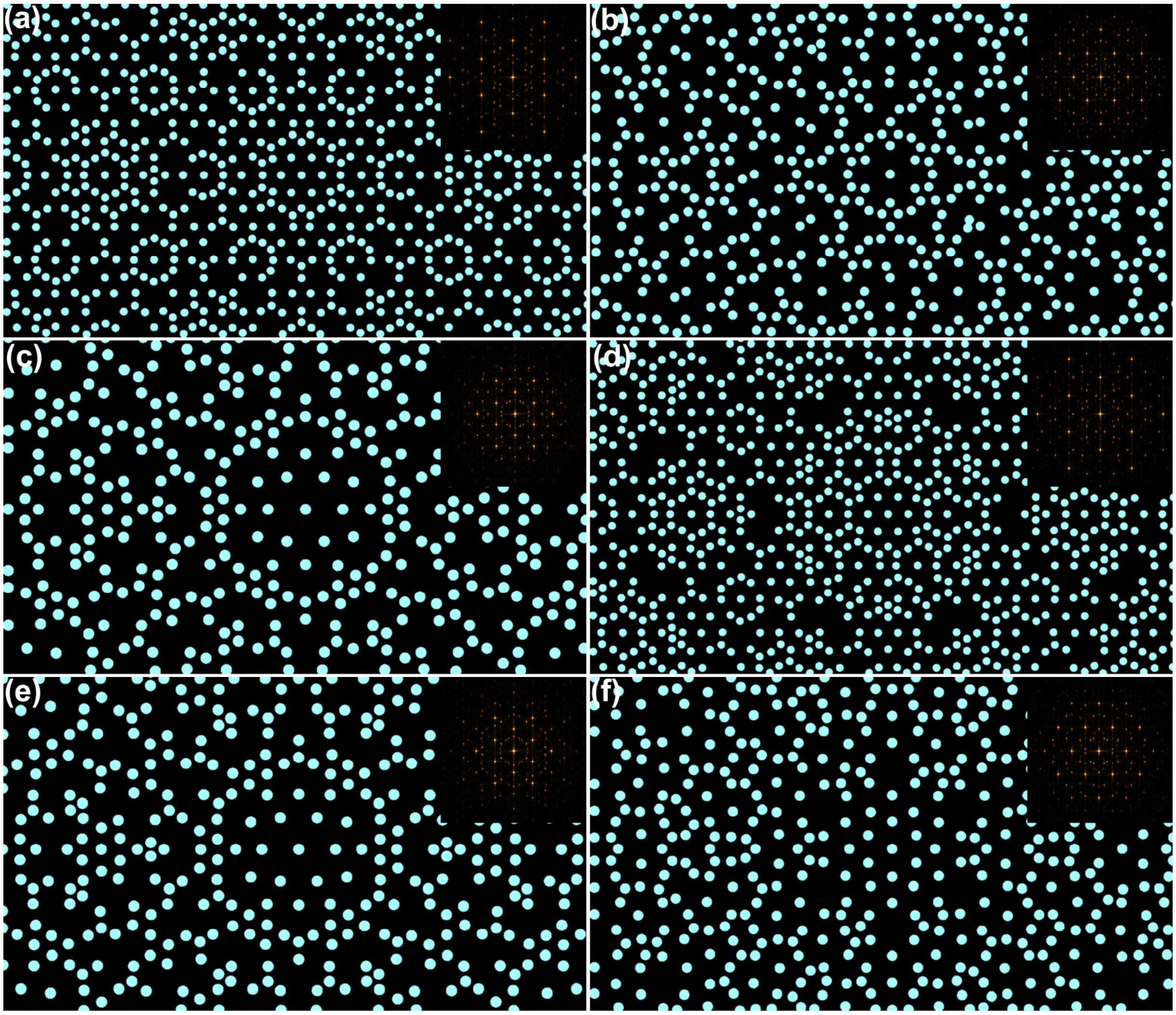

Figure S4

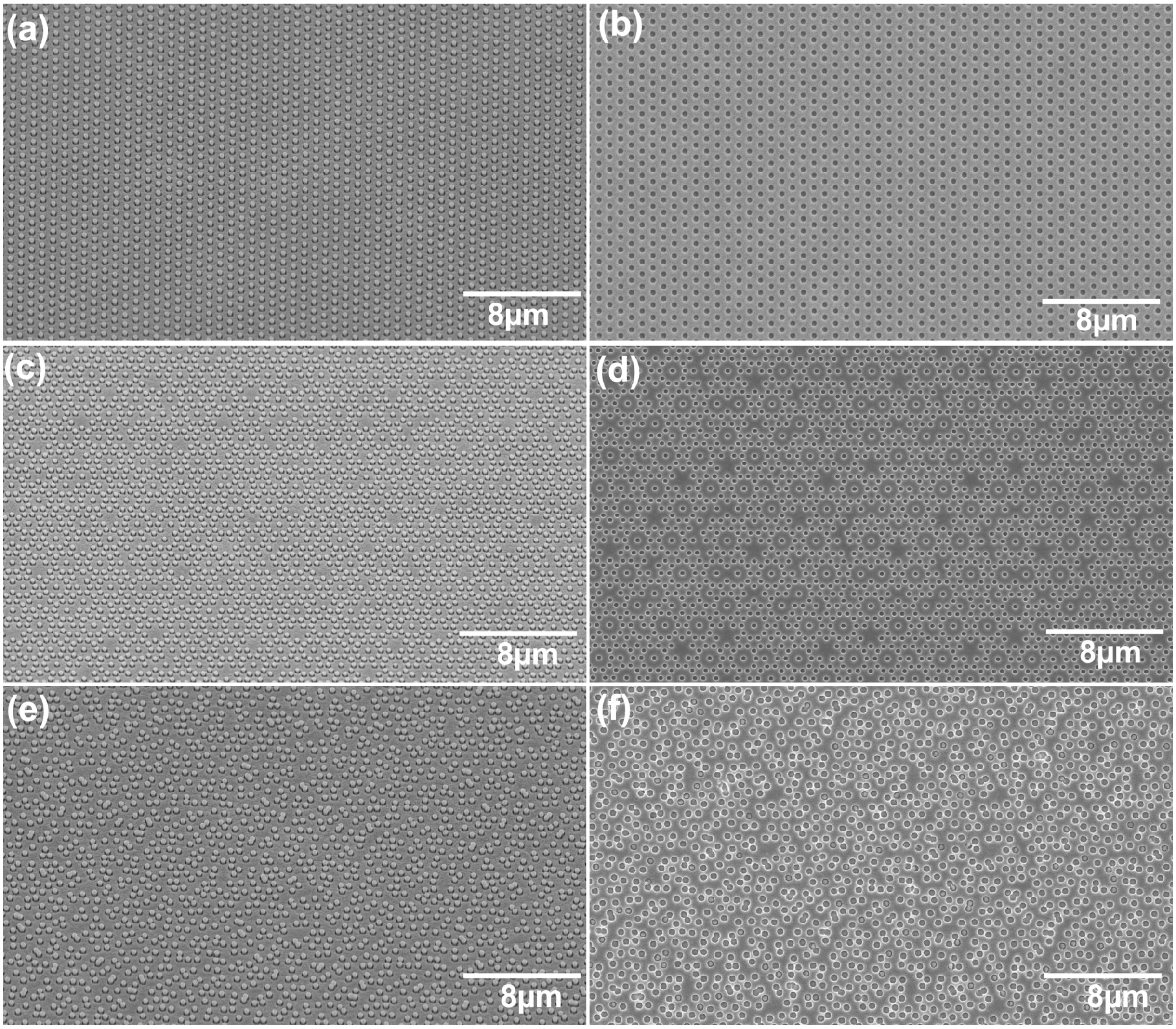

Supplement: Supplementary Information [file srep38744-s1.pdf]
